# Immune-related lncRNAs pairs prognostic score model for prediction of survival in acute myeloid leukemia patients

**DOI:** 10.1007/s10238-023-01085-2

**Published:** 2023-05-26

**Authors:** Xue Liang, Cong Li, Mengmeng Fan, Wanqiu Zhang, Linlin Liu, Ji Zhou, Linhui Hu, Zhimin Zhai

**Affiliations:** Department of Hematology/Hematological Lab, The Second Affiliated Hospital of Anhui Medical University, Hefei, Anhui China

**Keywords:** Acute myeloid leukemia, lncRNAs, Prognostic model, TGFβ, Chemoresistance

## Abstract

Acute myeloid leukemia (AML) is one of the most common malignant and aggressive hematologic tumors, and risk stratification is indispensable to ensure proper treatment. But immune-related long noncoding RNAs (ir-lncRNAs) pairs prognostic risk models used to stratify AML have yet to be reported. In this study, we established a prognostic risk model based on eight ir-lncRNAs pairs using LASSO-penalized Cox regression analysis and successfully validated the model in an independent cohort. According to risk scores, patients were divided into a high-risk group and a low-risk group. High-risk patients presented more tumor mutation frequency and higher expression of human leukocyte antigen (HLA)-related genes and immune checkpoint molecules. Gene Set Enrichment Analysis (GSEA) indicated that the transforming growth factors β (TGFβ) pathway was activated in the high-risk group; meanwhile, we found that TGFβ1 mRNA levels were significantly elevated in AML patients and correlated with poor prognosis, which is closely related to drug resistance. Consistently, in vitro studies found that exogenous TGFβ1 can protect AML cells from chemotherapy-induced apoptosis. Collectively, we developed an ir-lncRNA prognostic model that helps predict the prognosis of AML patients and provides valuable information about their response to immune checkpoint inhibitors, and we found that increased TGFβ1 levels resulting in chemoresistance may be one of the leading causes of treatment failure in high-risk AML patients.

**Supplementary Information:**

The online version contains supplementary material available at 10.1007/s10238-023-01085-2.

## Introduction

AML, the most common type of acute leukemia in adults, is a genetically heterogeneous clonal hematopoietic stem cell malignancy. During 2015–2019, the death rate was 2.7 per 100,000 men and women per year, and the five-year relative survival rate was 29.5% (The Surveillance Epidemiology and End Results (SEER) Program of the National Cancer Institute, Acute Myeloid Leukemia—Cancer Stat Facts). In this situation of high mortality, anthracycline- and cytarabine ( Ara-C)-based regimen still represents the primary chemotherapy, and the leading cause of AML-related mortality remains treatment failure due to refractory or relapsed disease [1]. Beyond molecular abnormalities and cytogenetic characteristics, the immune microenvironment also influences the prognosis of patients with AML [2]. Successful anti-cancer immunity relies on the capacity of effector immune cells to recognize and attack tumor cells and to alert other immune cells [3]. However, AML can create an immunosuppressive milieu, and innate and adaptive immune responses are profoundly deregulated [4].

With the rapid development of whole-transcriptome sequencing, thousands of noncoding RNAs have been unveiled; among them, longer than 200 nucleotides are defined as lncRNAs. lncRNAs regulate fundamental cellular processes, including transcription, RNA stability, and DNA methylation through interactions with proteins, other RNAs, and DNAs. Notably, lncRNAs could also regulate the function of the immune checkpoint [5]. Emerging evidence has identified that various ir-lncRNAs have significant associations with the cancer cell response to anti-PD-1 immunotherapy in multiple tumors [6, 7]. Some lncRNA signatures have been identified in AML, and their expression was associated with the survival of patients with AML [8–10]. Still, all these prognostic models were established based on the expression of lncRNAs, which limited the clinical application. Recently, a novel prognostic risk model based on ir-lncRNAs pairs used to stratify solid tumors has been reported, such as in breast cancer [11], hepatocellular carcinoma [12], and bladder cancer [13], but has not been investigated in AML.

TGFβ is a multipotent cytokine that regulates cell growth, differentiation, apoptosis, invasion, angiogenesis, matrix synthesis, immune homeostasis, and tolerance. The disorder in TGFβ signaling can induce inflammatory diseases and underlie tumor emergence. In tumor occurrence and development, TGFβ plays a dual tumor-suppressive and tumor-promoting role, which functions as a tumor suppressor that can induce apoptosis and inhibit proliferation during the initial carcinogenesis stages. However, it promotes tumorigenic, pro-metastatic responses and an immune-suppressive environment during tumor progression [14]. Furthermore, growing evidence suggests that TGFβ also correlates with increased resistance of carcinoma cells to chemotherapy in hepatocellular carcinoma [15], colorectal cancer [16], and squamous cell carcinoma [17].

In this study, we used the BeatAML public database to determine major ir-lncRNAs pairs and built a new prognostic model using LASSO-penalized Cox regression analysis. Further exploration of the correlations between clinical traits and identified risk groups was performed. Furthermore, we predicted patients in the high-risk group might profit from immune checkpoint treatment that could provide a reference to establish more rational treatment strategies. Meantime, GSEA was used to analyze the enriched KEGG pathway, and the TGFβ signaling pathway was found to increase in high-risk patients. Then, we explored whether the TGFβ pathway played a critical role in maintaining the malignant phenotype of AML cells in vitro. We discovered that TGFβ1 mRNA levels were associated with a poor clinical prognosis, and exogenous TGFβ1 protected AML cells from chemotherapy-induced apoptosis. These results revealed that activating the TGFβ pathway could predispose an adverse consequence via drug resistance.

## Methods

### Data source

The training data series was retrieved from BeatAML database (http://www.vizome.org/aml/) and consisted of RNA sequencing data and corresponding detailed clinical annotations, including diagnostic information, treatments, responses, and outcomes of 242 AML patients [18].

The testing dataset was downloaded from The Cancer Genome Atlas (TCGA) database (https://portal.gdc.cancer.gov/) and consisted of transcriptional profiles, and the corresponding clinical and overall survival (OS) data of 173 AML patients.

### Construction of ir-lncRNAs pairs

First, a total of 2,483 immune-related genes were collected from the ImmPort Database (https://immport.niaid.nih.gov), then the relationship between all lncRNAs and immune-related genes was indicated by correlation tests, and the highly correlated lncRNAs were considered as ir-lncRNAs (R > 0.5 and P value of < 0.05, Supplementary Table S1). Next, differential expression analysis was performed and significant ir-lncRNAs were chosen with the fold change > 1 and FDR < 0.05. Finally, ir-lncRNAs were paired tautologically, if the expression of lncRNA A is higher than that of lncRNA B, the value is defined as 1, if not, the value is 0; then, the 0 or 1 matrix was established.

### Establishment of a prognostic model

After combining the 0 or 1 matrix profile with the prognosis data, we performed univariate cox analysis and objects that were statistically significant (p-values < 0.05) in this univariate analysis were then used for LASSO-penalized Cox regression analysis. As a result, a risk score model including eight ir-lncRNA pairs was built. Then, the univariate and multivariate analysis was performed to demonstrate the independence of each ir-lncRNAs pair in this model. Eventually, the risk score of each patient was calculated by integrating the regression coefficients and ir-lncRNAs pair levels. The predictive capacity of risk model was validated by Kaplan–Meier (K–M) survival analysis using the following R packages: “survival,” “glmnet,” “pbapply,” “survivalROC,” and “survminer.” Time-dependent receiver operating characteristic (ROC) curves with AUC values were used to evaluate the ability of prognosis classification.

### Human samples, RNA isolation and real-time PCR analysis

Samples were isolated from residual bone marrow samples after laboratory tests of patients newly diagnosed with AML (n = 33) or IDA (n = 15) from April 2019 to April 2021 at the Second Hospital of Anhui Medical University, with no congenital/acquired immunodeficiency, were enrolled, according to the Chinese guidelines for diagnosis and treatment of AML (2021). All human samples used were obtained under the approval of the Ethics Committee of the Second Hospital of Anhui Medical University. All patients enrolled in the study signed informed consent. Leucocytes were enriched by hemolysis of erythrocytes, and total RNA was isolated by Trizol extraction, followed by reverse transcription. Then, the expression of TGFβ1 and TGFβ receptor 2 (TGFβR2) was analyzed by Applied Biosystems 7500 Real-time Polymerase Chain Reaction (PCR) (Life, Grand Island, NY, USA). TGFβ1 primers were as follows: 5’- CAATTCCTGGCGATACCTCAG (sense) and 5’-GCACAACTCCGGTGACATCAA (anti-sense). TGFβR2 primers were as follows: 5’-TGTTGAGTCCTTCAAGCAGACCGA (sense) and 5’-ACTTCTCCCACTGCATTACAGCGA (anti-sense).

### Cell line and cell culture

AML cell lines KG-1a was ordered from the Shanghai cell bank of the Chinese Academy of Sciences and cultured in RPMI 1640 (Gibco) supplemented with 20% fetal bovine serum. Cells were maintained at 37 °C in a 5% CO_2_ incubator. KG-1a was precultured with 10 ng/ml TGFβ1 (PeproTech) for 48 h and used for Cell Counting Kit-8 (CCK8) assay (Beyotime Institute of Biotechnology, China), apoptosis detection (Apoptosis Detection Kit, BestBio), cell cycle detection (COULTER DNA PREP Reagents Kit, Beckman). Cell cycle phase distributions were generated by fitting DNA content histograms to applicable models using ModFit LT software for Windows (Version 5.0). KG-1a cells were stained with Senescence‐associated‐β‐Galactosidase (SA‐β‐Gal) staining (Beyotime) according to the instructions after TGFβ1 treatment for 7 days, and cells that stained green‐blue were evaluated as positive senescent cells. Cell suspension (1 × 10^5^, 200 μl of serum-free medium) after TGFβ1 pretreatment was seeded onto 8-mm Pore Transwell Inserts (Corning) in 24-well plates with or without matrigel (BD biosciences) and photographed within 24 h.

### Statistical analysis

SPSS software vision 25.0 (SPSS, Inc., Chicago, IL, USA) and R software vision 4.2.2 (R Foundation for Statistical Computing, Vienna, Austria) analyzed the data in statistics. P values less than 0.05 were considered to be statistically significant.

## Results

### Establishing an ir-lncRNAs pairs prognostic risk model for AML patients

**Fig. 1:**
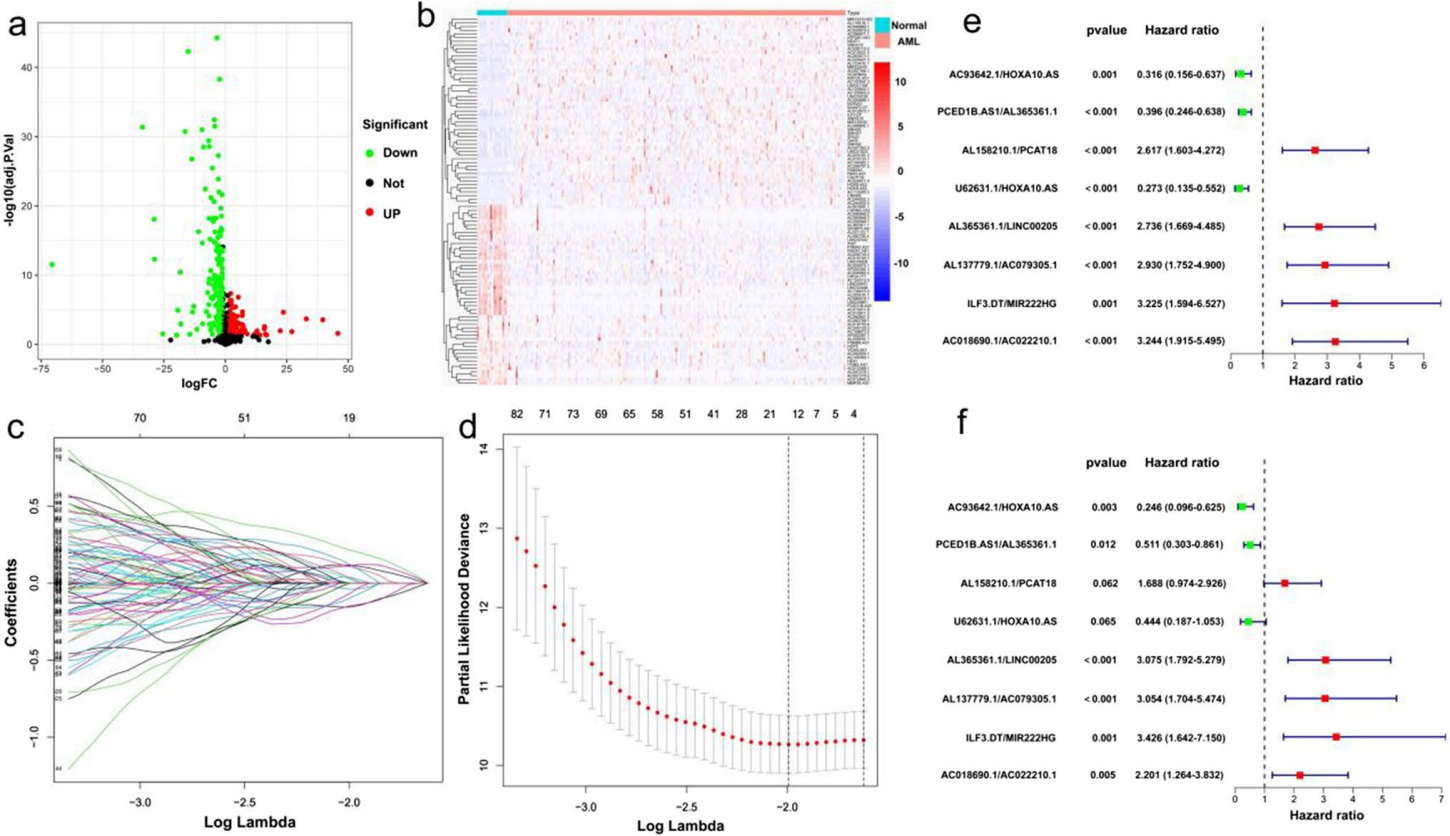
Construction of the ir-lncRNAs pairs prognostic model. (**a**) Differentially expressed lncRNAs in AML and normal tissues. (**b**) Heatmap of differential lncRNAs. (**c**) The LASSO coefficient values at various levels of penalty; each curve represents an ir-lncRNAs pair. (**d**) The confirmation of the best lambda value by LASSO Cox regression analysis. (e, f) Univariate and multivariate Cox regression analysis of the each pairs in this model

The expression profiles of 1289 ir-lncRNAs in BeatAML were used for differential expression analysis (Fig. 1a). There were 380 differentially expressed genes in AML and normal tissues, of which 170 were upregulated and 210 were downregulated in AML(Fig. 1b). Then, we constructed a 0 or 1 matrix to generate ir-lncRNAs pairs based on the above 380 genes and 16,381 pairs were identified using univariate analysis. Then, 1710 ir-lncRNAs pairs were verified by LASSO-penalized Cox regression for screening the prognosis-related genes with potentiality. Figure 1c shows the coefficient values for each at various penalty levels as long as ir-lncRNAs pairs with nonzero coefficients had prognostic values in the LASSO-penalized regression model. Tenfold cross-validation obtained the maximum lambda value, and we selected one model which produced a group of eight pairs(AC93642.1|HOXA10.AS, PCED1B.AS1|AL365361.1, AL158210.1|PCAT18, U62631.1|HOXA10.AS, AL365361.1|LINC00205, AL137779.1|AC079305.1, ILF3.DT|MIR222HG, AC018690.1|AC022210.1, Fig. 1d). Finally, the 8 ir-lncRNAs pairs selected by the LASSO-penalized regression model were evaluated for their survival risk through univariate and multivariate Cox proportional hazard regression analyses, comprehensively investigating the relationship between the 8 ir-lncRNAs pairs and the prognosis of AML. The univariate analysis suggested that AC93642.1|HOXA10.AS, PCED1B.AS1|AL365361.1, and U62631.1|HOXA10.AS were *protective* factor (Hazard ratio < 1), while the other five pairs might have hazardous effects (Hazard ratio > 1, Fig. 1e**)**. Similarly, we found that AC93642.1|HOXA10.AS, PCED1B.AS1|AL365361.1, AL365361.1|LINC00205, AL137779.1|AC079305.1, ILF3.DT|MIR222HG, AC018690.1|AC022210.1 were independent prognostic factors (p < 0.05, Fig. 1f). We first identified the differentially expressed ir-lncRNAs between the normal control and AML patients in BeatAML and constructed ir-lncRNAs pairs from these differentially expressed ir-lncRNAs. Then, we screened out 8 prognosis-related ir-lncRNAs pairs through LASSO regression analysis and evaluated the survival risk of these 8 ir-lncRNAs pairs through univariate and multivariate analysis.

### Patients classified as high-risk by the ir-lncRNAs pairs prognostic risk model have a worse prognosis

**Fig. 2:**
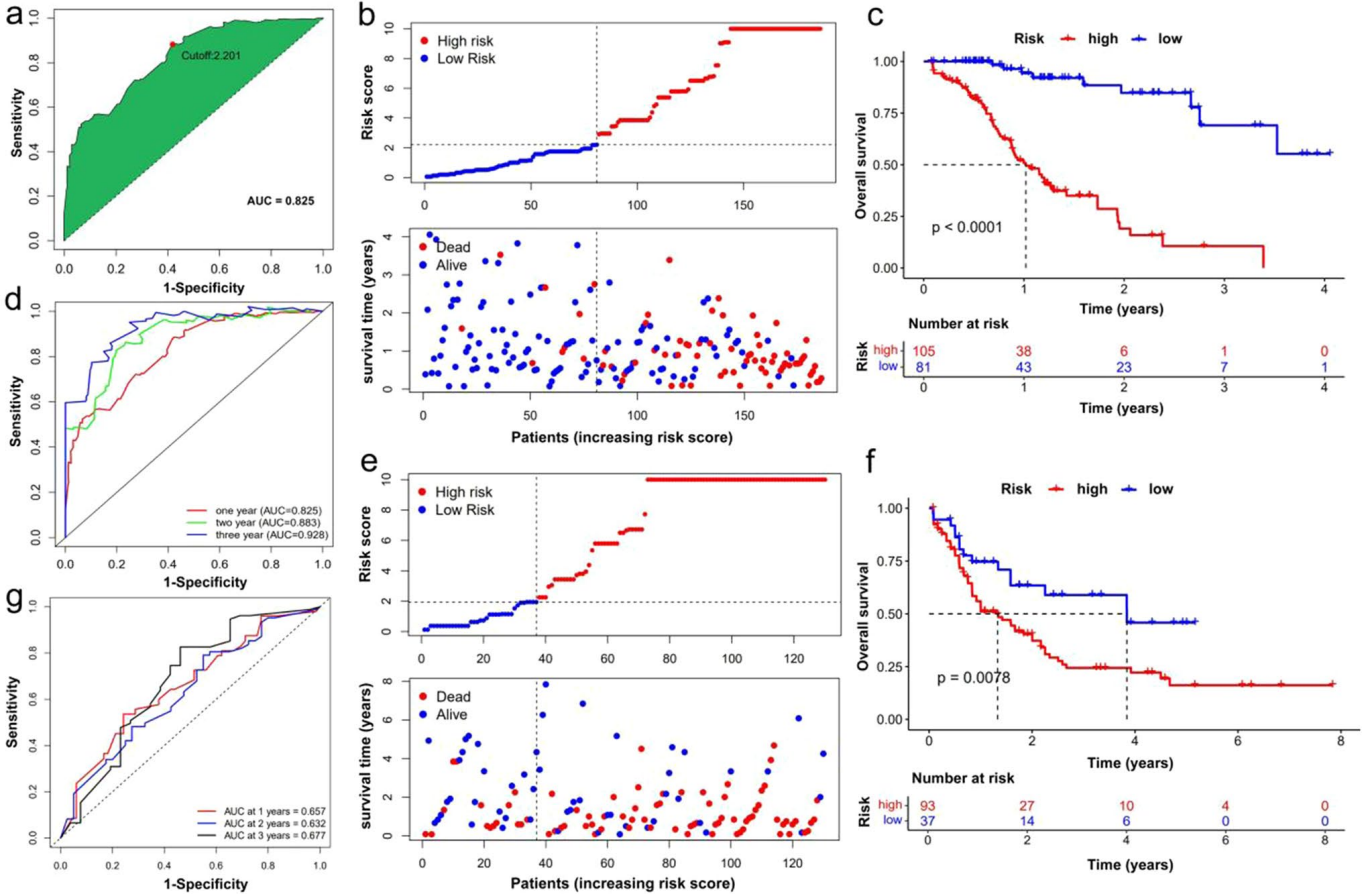
Patients classified as high-risk group in the train and test columns have poorer prognosis. (**a**) AUC of 1-year ROC curve and confirmation of cutoff value. (**b**) Dot plots comparing the outcomes of subjects and survival time in the high- and low-risk groups of BeatAML database. (**c**) Survival curves of two groups in BeatAML database were plotted using the K–M method. (**d**) Time-dependent ROC curve analysis for the prediction survival using the risk model in BeatAML database. (**e**) Dot plots comparing the outcomes of subjects and survival time in the high- and low-risk groups of TCGA database. (**f**) Survival curves of two groups in TCGA database were plotted using the K–M method. (**g**) Time-dependent ROC curve analysis for the prediction survival using the risk model in TCGA database

First, the AUCs of the 1-year ROC curve for the ir-lncRNAs pairs was 0.825, and the optimal cutoff value was 2.201 (Fig. 2a). Then, the patients from the BeatAML database were divided into high-risk and low-risk groups based on the cutoff value, and each patient's risk score and survival data are shown in Fig. 2b. The results confirmed that death was more frequently observed in the high-risk group than in the low-risk group. The K–M survival analysis presented a much worse outcome in the high-risk group than in the low-risk group (log-rank test, p < 0.0001, Fig. 2c). Also, the AUCs of a time-dependent ROC curve of 1, 2, and 3 years calculated by the risk score model were 0.825, 0.883, and 0.928, respectively (Fig. 2d), suggesting that the prediction was highly sensitive and specific.

The testing column (TCGA database) verified the predictive values of the eight ir-lncRNAs pairs risk score. Patients were also divided into low-risk and high-risk groups based on the cutoff value, and each patient's risk score and survival data are shown in Fig. 2e. The K–M curve promoted patients classified to these two level risk scores obtained a different OS (log-rank test, p = 0.0078, Fig. 2f). The AUCs of 1-, 2- and 3-year ROC curves were 0.657, 0.632, and 0.677 (Fig. 2g). These results showed that the ir-lncRNAs pairs prognostic model might be a potential predictor to judge the OS of patients with AML.

### Relationship between clinical factors and ir-lncRNAs pairs risk model

**Fig. 3:**
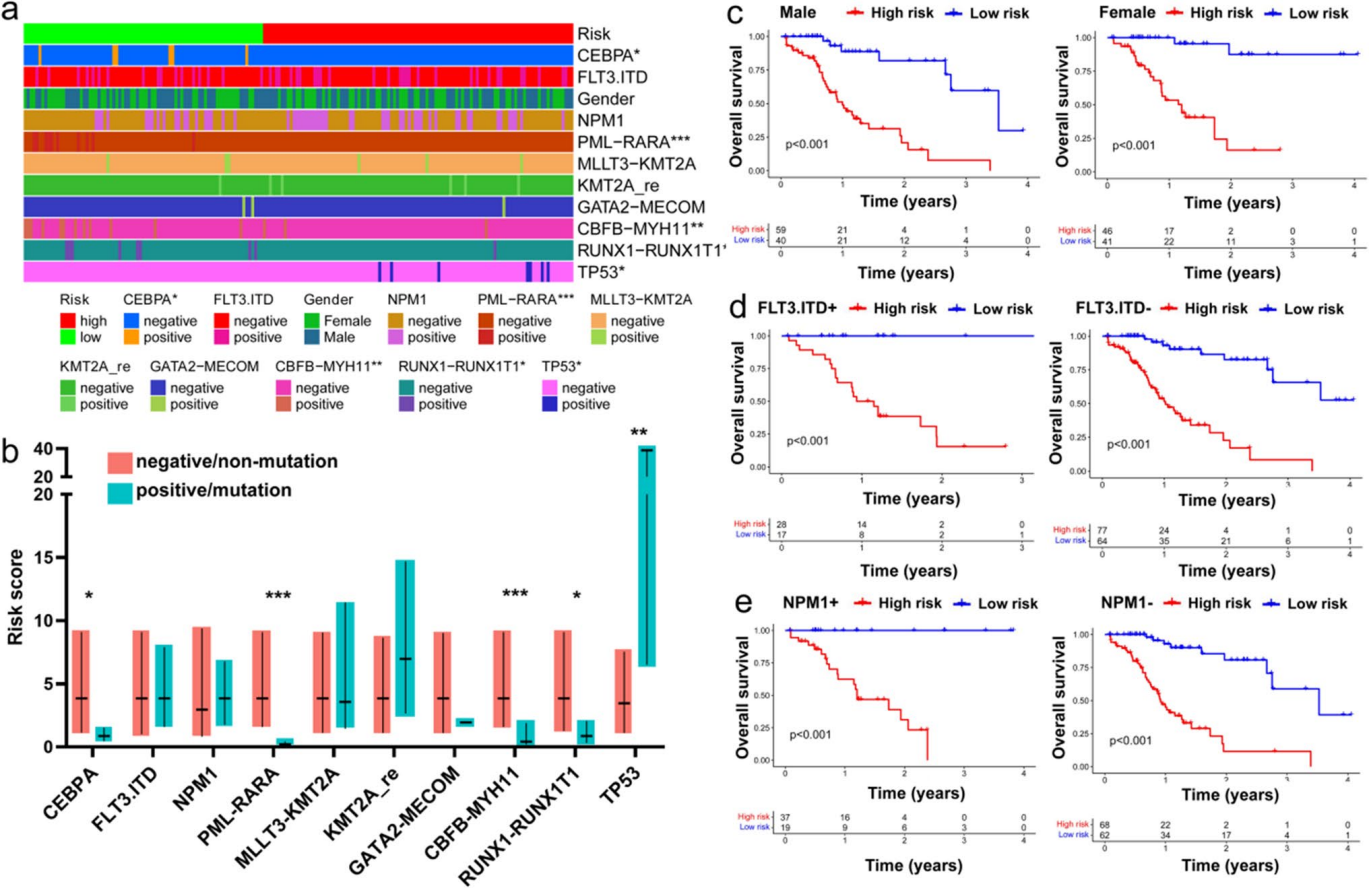
Relationship between clinical factors and ir-lncRNAs pairs risk model. (**a**) Differences in clinical characteristics between high- and low-risk groups (Chi-squared analysis). (**b**) A boxplot comparing the risk scores of each patient across clinical characteristics, with boxes showing first and third quartiles and medians (Wilcoxon test). (**c**-**e**) K–M survival curves showing the subgroup analyses of gender, NMP1 and FLT3 mutation status. KMT2A-re, KMT2A-rearrangement; NPM1 + , NPM1-mutated group; NPM1-, NPM1-unmutated group

AML is characterized by heterogeneous genetic abnormalities, and the differences in the frequency distribution of genetic abnormalities and gender between high and low-risk groups were determined by Chi-squared analysis. The strip chart showed that the incidence of CEBPA mutation, PML-RARα, CBFB-MYH11, and RUNX1-RUNX1T1 was higher in the low-risk group, and the reverse situation was observed for TP53 mutation (Fig. 3a). Meanwhile, we compared the risk score of different groups. We found that CBFB-MYH11, PML-RARα, RUNX1-RUNX1T1 positive group, and CEBPA mutated group had lower risk scores than the fusion proteins negative group and no mutation group. Still, the risk score of patients with TP53 mutation was higher than patients without mutation (p = 0.002). In the remaining groups, no difference was observed (Fig. 3b). Finally, the K–M survival curve for OS in different subgroups was performed. Since the number of positive cases in CEBPA mutation, TP53 mutation, PML-RARα, MLLT3-KMT2A, KMT2A-rearrangement, GATA2-MECOM, CBFB-MYH11, and RUNX1-RUNX1T1 was too small, the survival analysis was not conducted. Subsequently, AML patients were divided into subgroups according to gender, NPM1 mutation status, and FLT3-ITD mutation status, respectively, and KM analysis was further performed in each subset. The results showed that the OS of patients with high-risk was significantly shorter than that with low-risk regardless of the FLT3-ITD, NPM1 mutation status, or gender (Fig. 3c–e).

### Differences in mutational profiling and immune landscape between high- and low-risk groups

**Fig. 4:**
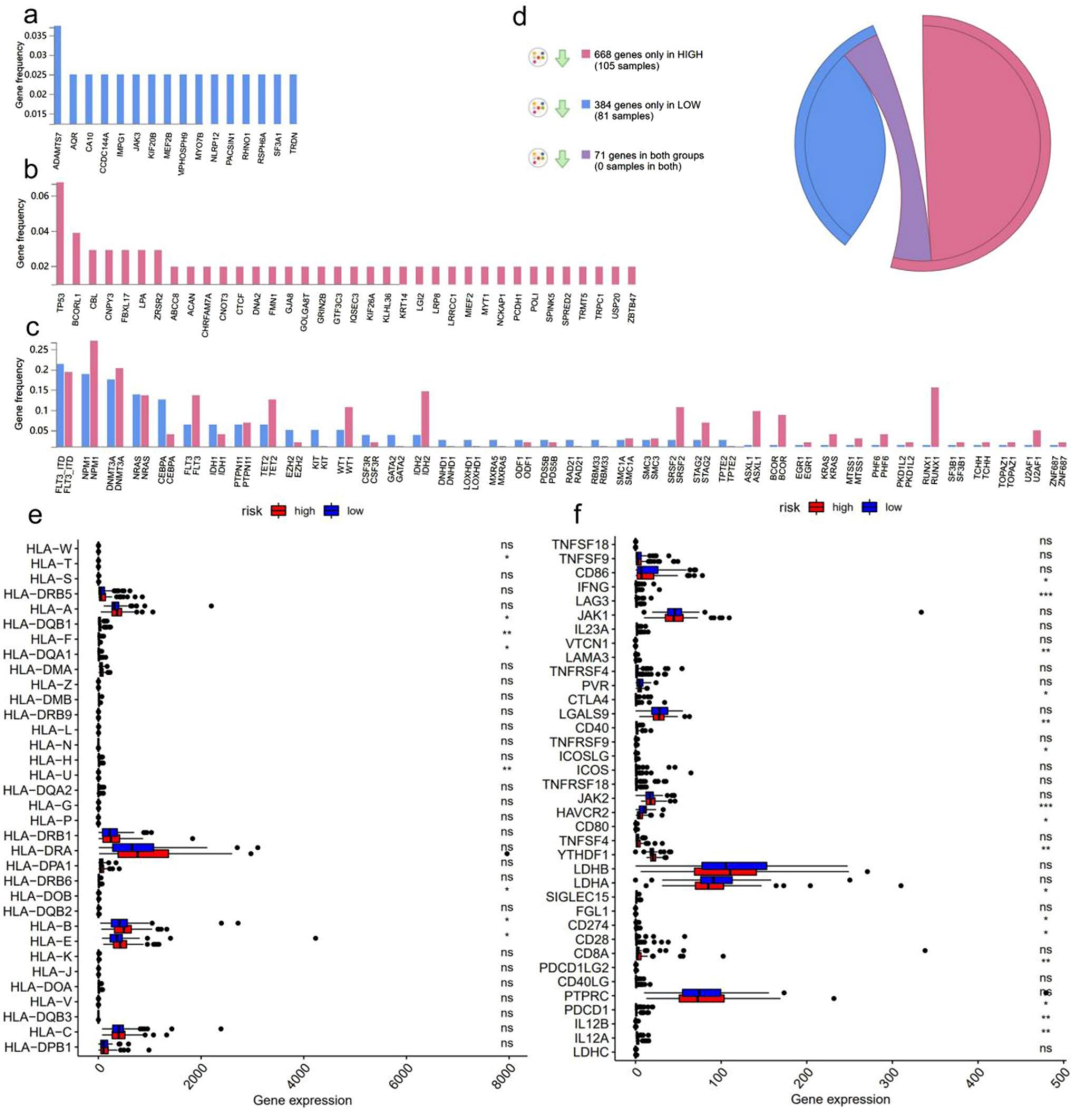
Mutational profiling and immune landscape between high- and low-risk groups. (**a**-**d**) Gene mutation spectrum differences between high-risk and low-risk patients in the BeatAML database. (**e**, **f**) Differential expression of HLA-related genes and immune checkpoint genes between high- and low-risk patients in BeatAML database

First, we analyzed the mutational profiling of the high- and low-risk groups in the BeatAML database using an online website (www.vizome.org/aml/) (Fig. 4a–c**)**. Figure 4a and b shows the spectrum of mutated genes occurring only in the low- and high-risk groups, respectively, while Fig. 4c shows the mutated genes co-occurring in the high- and low-risk groups; 384 genes mutation occurred only in the low-risk group, 668 genes mutation occurred only in the high-risk group, and 71 genes mutation occurred in both groups (Fig. 4d).

Next, we compared the expression of 34 HLA-related genes between high- and low-risk patients and found that HLA-B, HLA-E, HLA-F, HLA-T, HLA-U, HLA-DOB, HLA-DQA1, and HLA-DQB1 were significantly upregulated in the high-risk group (Fig. 4e). In addition, the expression of 37 immune checkpoint molecules was detected between the high-risk and low-risk groups, 14 of which were upregulated in high-risk patients, including CTLA-4, PDCD1, CD274, LAG3, and PDCD1LG2, among others (Fig. 4f). These results suggested that high-risk patients may have better responses and better outcomes when receiving immune checkpoint inhibitors.

### Elevated mRNA levels of TGFβ1 are associated with poor prognosis in AML patients

**Fig. 5:**
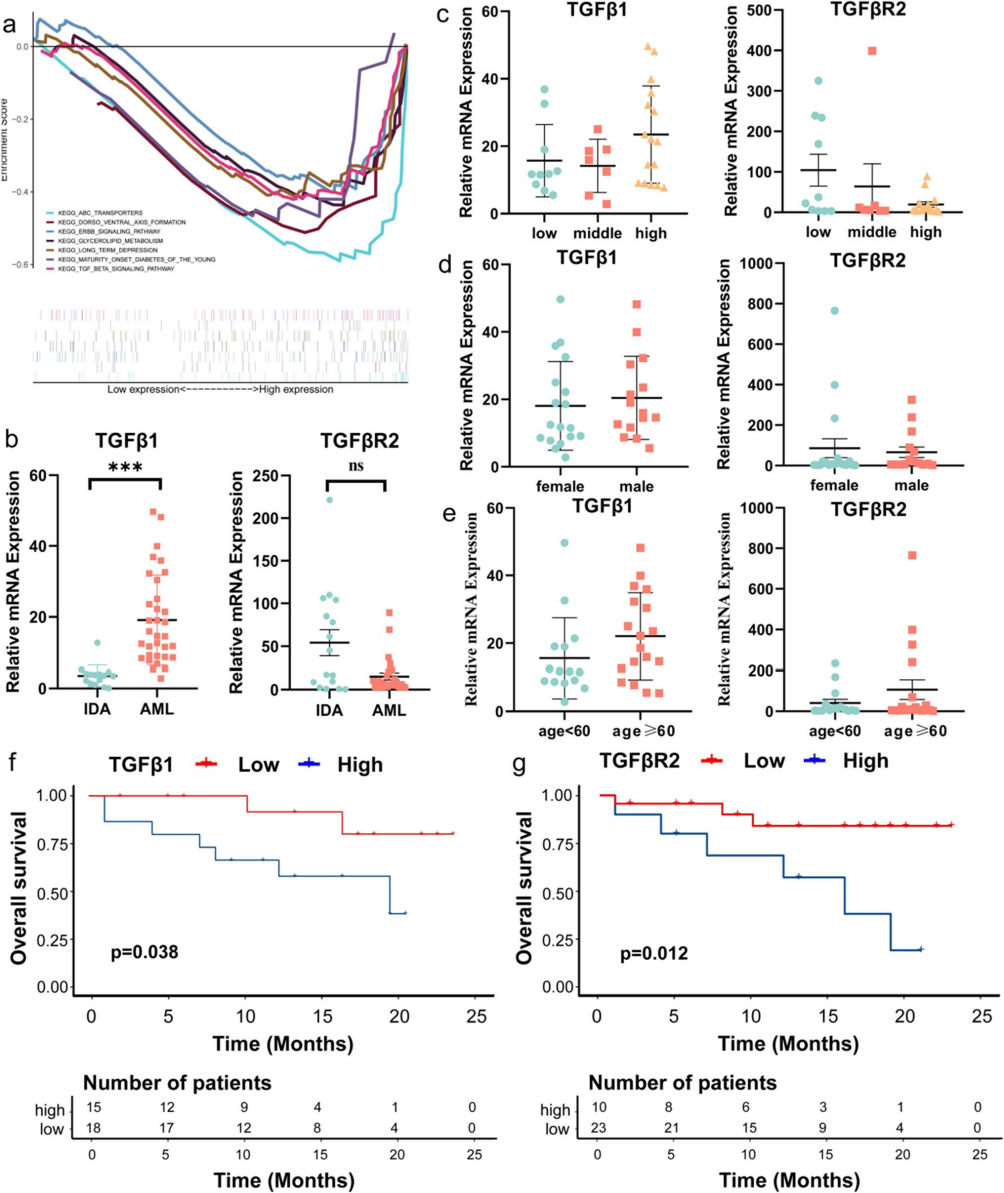
TGFβ1 mRNA levels are elevated in AML patients and associated with poor prognosis. (**a**) GSEA revealed that seven signaling pathways were enriched in high-risk patients. (**b**) The relative expression of TGFβ1 and TGFβR2 mRNA in the bone marrow of patients with AML and IDA. (**c**-**e**) The comparison of different risk stratification, gender, and ages, respectively. (**f**, **g**) K–M analyses of overall survival rates of AML patients according to TGFβ1 and TGFβR2 expression levels

GSEA was performed to compare the differences in enriched signaling pathways between the high- and low-risk groups, and the results suggested that seven signaling pathways were enriched in the high-risk group, including ABC transporters, TGFβ signaling pathway, dorsoventral axis formation, ERBB signaling pathway, glycerolipid metabolism, long-term depression, and maturity onset diabetes of the young (|NES|> 1, NOM p < 0.05, Fig. 5a). High-risk patients usually have a poor prognosis, and the poor prognosis of AML is closely related to drug resistance, so we considered whether TGFB is related to drug resistance.

To investigate the expression of TGFβ1 and its receptor TGFβR2 and to examine whether their expression correlates with disease characteristics and clinical outcomes in adult patients with de novo AML, we collected 33 AML and 15 IDA bone marrow biopsy specimens. In our study, the AML group showed elevated TGFβ1 mRNA levels compared to the IDA control group (*p* < 0.001); conversely, TGFβR2 was downregulated in AML patients (*p* = 0.09, Fig. 5b). Furthermore, a simple comparison of TGFβ1 and TGFβR2 mRNA levels between male and female patients showed no significant difference, neither in age nor risk stratification (Fig. 5c–e). Results of univariate and multivariate Cox proportional hazards regression analysis of TGFβ1, TGFβR2, and other clinical indicators are shown in Table 1. The high expression of TGFβR2, TGFβ1, and low hemoglobin levels were considered risk factors in AML patients. Last, the survival curves of TGFβ1 and TGFβR2 were plotted using the K–M method. Patients were divided into high- and low-level groups according to the cutoff value based on ROC curves, and we found the patients in the low-level TGFβ1 and TGFβR2 group had a better survival prognosis (Fig. 5f, g). These results suggested that TGFβ expression was upregulated in AML patients and was associated with poor prognosis.

**Table 1:** Univariate and multivariate Cox regression analysis of clinical traits, TGFβ1 and GFβR2

	Univariate analysis			Multivariate analysis		
	HR	95%CI	p	HR	95%CI	p
BLAST	0.994	0.966–1.022	0.664			
WBC	0.986	0.962–1.010	0.243			
HB	0.973	0.938–1.008	0.129	0.924	0.872–0.980	0.008*
PLT	1.006	0.990–1.023	0.438			
AGE	1.018	0.981–1.056	0.334			
SEX	1.532	0.133–2.139	0.374	0.239	0.044–1.291	0.096
ALB	0.912	0.771–1.080	0.286			
TGFβ1	1.017	0.973–1.064	0.446	1.104	1.004–1.213	0.142*
TGFβR2	1.004	1.001–1.007	0.009*	1.005	1.003–1.009	0.033*
RISK	1.187	0.567–2.484	0.650			

### Exogenous TGFβ1 resists AML cells to chemotherapy.

**Fig. 6:**
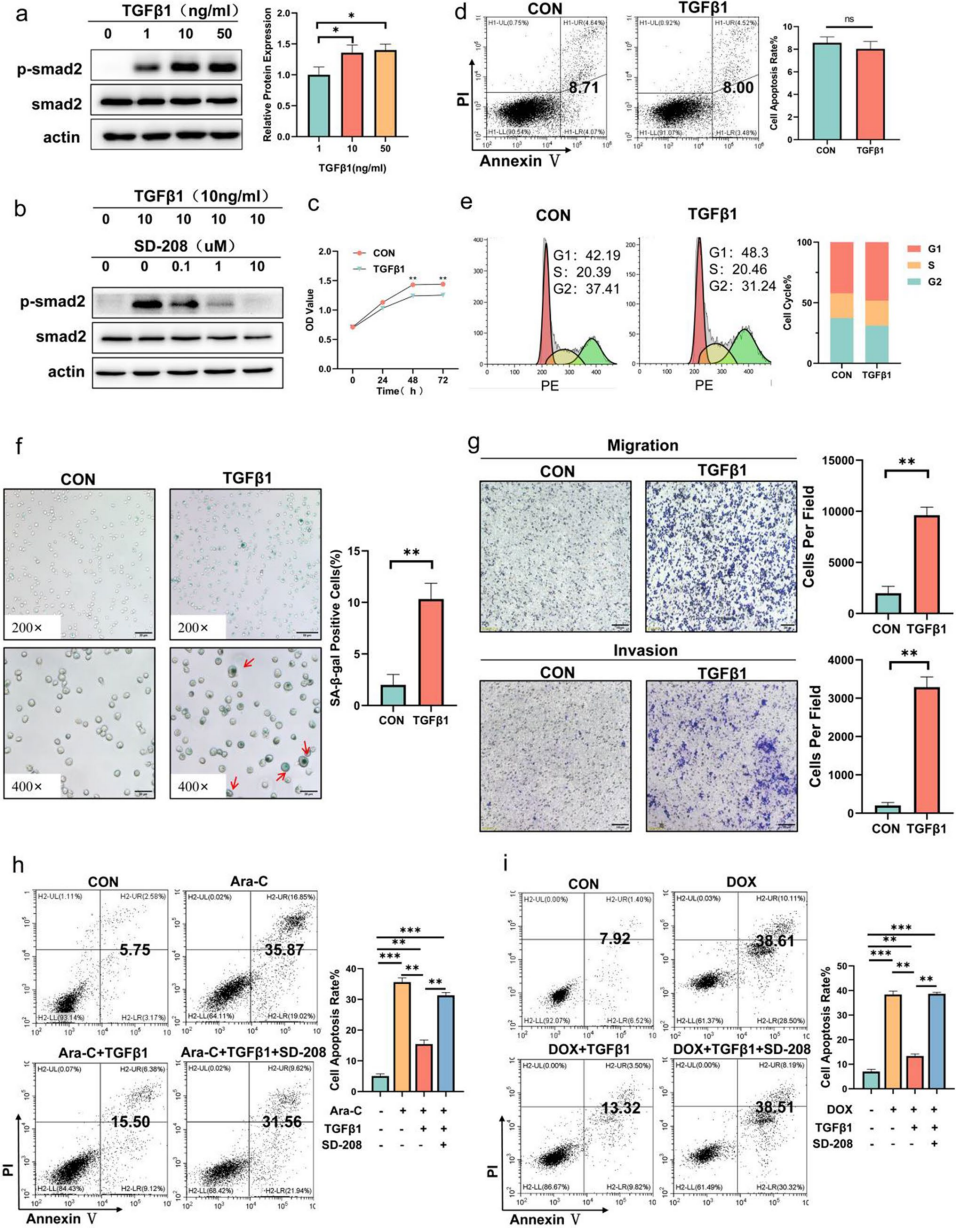
Exogenous TGFβ1 resists chemotherapy in AML cells. (**a**) Phosphorylation of downstream Smad2 gradually increased with increasing concentration of exogenous TGFβ1. (**b**) Small molecular TGFβR1 inhibitors SD-208 inhibited smad2 phosphorylation by TGFβ1. (**c**) CCK8 results showing the effect of TGFβ1 on the proliferation of KG-1a at 24, 48 and 72 h. (**d**, **e**) Flow cytometry showing the effect of TGFβ1 on KG-1a apoptosis and cell cycle. (**f**) SA‐β‐Gal staining showing the rate of senescence cell after TGFβ1 treatment for 7 days. (**g**) Transwell migration and matrigel invasion assays showing the increasing ability of motility and invasion after TGFβ1 treatment. (**h**, **i**) Flow cytometry data indicating the percentage of apoptotic cells co-cultured for 48 h with Ara-C and DOX pre-treated with DMSO, TGFβ1, and TGFβ1 plus SD-208

We then performed experiments to determine whether exogenous TGFβ1could enhance the malignancy of AML cells, especially drug resistance. KG-1a cells, human leukemia progenitors cell line which could respond to TGFβ1, were selected as a cell model for subsequent experiments. To determine the optimum concentration of exogenous TGFβ1, we set a series of concentration gradients for 48 h, and 10 ng/ml TGFβ1 was used for subsequent experiments based on the smad2 phosphorylation levels (Fig. 6a). Simultaneously, this kind of phosphorylation could be inhibited by one of small molecular TGFβR1 inhibitors named SD-208 when the concentration was 10 uM (Fig. 6b).

We next assessed the effect of TGFβ1 on KG-1a primary biological function and found exogenous TGFβ1 slightly suppressed the proliferation of KG-1a, and this inhibition was due to cell cycle arrest instead of apoptosis (Fig. 6c–e). Besides, the percentage of senescence cells and cells crossing a transwell membrane increased after TGFβ1 treatment (Fig. 6f, g). To determine whether TGFβ1 could protect AML cells from the pro-apoptotic effect of cytarabine and doxorubicin (DOX), KG-1a cells with or without TGFβ1 pretreatment were incubated with 50uM Ara-C and 3uM DOX, respectively. We observed that the proportion of apoptotic cells was attenuated after TGFβ1 pretreatment; notably, this phenomenon could be reversed by SD-208 (Fig. 6h, i). TGFβ1 can increase the degree of malignancy by promoting the senescence, migration, invasion, and anti-apoptosis ability of KG-1a cells.

## Discussion

During the development and progression of AML, malignant cells evolve and may exhibit a series of mechanisms to evade tumor immunosurveillance and suppress anti-tumor immune responses [19]. The tumor microenvironment profile impacts the efficacy of anti-cancer therapies and has a significant impact on prognosis [20]. A small number of lncRNAs have been discovered and characterized as immune regulators and reported to regulate the tumor microenvironment by targeting genes implicated in the function of immune cells [21]. Hence, we constructed an ir-lncRNAs pairs prognostic model to refine risk stratification and ensure proper treatment. The previous prognostic model mostly used gene expression, which made it difficult to apply directly to clinical genetic testing. Here, we used ir-lncRNAs pairs to obtain a relative expression value to make it possible to apply this model to the clinic.

The prognostic value of the ir-lncRNAs pairs risk model was verified in both a training set and an independent validation set, and patients were separated into low- and high-risk groups. In this study, the highest mutation frequency gene only occurring in high-risk patients was TP53, which activated transcription of critical regulators of the hematopoietic stem cell functions. Dysregulation of pathways downstream of mutated TP53 may mediate resistance to chemotherapy [22]. According to the last updated AML classification, MDS, MDS/AML, and AML with mutated TP53 are grouped together due to their overall similar aggressive behavior irrespective of the blast percentage [23]. In MDS, the presence of multi-hit TP53 mutations corresponds to a highly aggressive disease with short survival, and monoallelic TP53 mutations have a less adverse effect on prognosis. However, monoallelic mutated TP53 MDS/AML and AML already have a poor prognosis. Similarly, we found that the risk scores of patients with TP53 mutation were higher than those without mutation (*p* = 0.002). The most predominant mutation form was the missense variant, followed by frameshift variant, and splice variant. For those co-occurring genes in both high- and low-risk groups, IDH2, ASXL1, and RUNX1 demonstrated higher mutation frequency in the high-risk group, and the mutation of CEBPA, which portends a favorable outcome, was more prevalent in the low-risk group.

Multiple factors influence the immune response against tumors, including tumor cell intrinsic and extrinsic mechanisms. Absence of antigenic proteins, absence of antigen presentation, genetic T cell exclusion, and insensibility to T cells belong to intrinsic reasons. And absence of T cells, inhibitory immune checkpoints, and immunosuppressive cells are the extrinsic mechanisms [23].

HLA is a major histocompatibility complex (MHC) closely related to human immune system function, which involves the process of T cell activation and varies significantly from person to person. Several factors may influence antigen recognition and presentation, including antigen expression level, diversity, and ability. Patients with decreased expression of HLA-I( HLA-A, HLA-B, and HLA-C), HLA homozygosity and somatic loss of heterozygosity (LOH), and the inability of patient-specific MHC-I complexes to bind and present neoantigens, have a lower efficiency of antigen delivery and poor survival to immune checkpoint blockade therapy [24]. In our research, high-risk patients had a higher expression of 9 HLA-related genes than low-risk patients, which means the former might have stronger and sustained antigen presentation capacity.

On the other hand, 14 checkpoint genes were upregulated in high-risk patients, including CTLA-4, PDCD1, CD274, LAG3, and PDCD1LG2. Such patients were also more likely to benefit from immune checkpoint inhibitors. Recent studies have reported that in NSCLCs treated with pembrolizumab, an antibody targeting PD-1, an elevated nonsynonymous mutation burden in tumors is associated with improved objective response, durable clinical benefit, and progression-free survival [25]. This result suggests that a high mutation burden may help predict the response to immune checkpoint therapy [26]. Interestingly, high-risk patients had more mutated genes than low-risk patients in our research. In combination, the above results support the idea that high-risk patients may benefit more from immunotherapy.

In our research, GSEA suggested that seven signaling pathways were enriched in the high-risk group. The ABC transporters, ERBB signaling pathway, and glycerolipid metabolism are associated with drug resistance, and their role in AML resistance has been widely studied [27–30]. However, studies on the TGFβ signaling pathway in AML drug resistance are relatively limited. Therefore, further studies on this pathway may help to reveal the mechanisms of AML drug resistance and to develop more effective treatment strategies for AML. Interestingly, clinical data indicated that the TGFβ1 mRNA level in bone marrow of newly diagnosed AML was higher than in IDA patients, but TGFβR2 showed the opposite trend. Both TGFβ1 and TGFβR2 remained two significant risk factors and predicted worse survival. Previous studies have demonstrated that the inhibitory effect of TGFβ1 on the growth of human myeloid and lymphoid cells revealed that most TGFβ-insensitive AML cell lines had no detectable TGFβ receptor, while the rest had reduced numbers of receptors [31]. However, the opposite effect of TGFβ has also been reported; for example, TGFβ derived from activated bone marrow microenvironment enhanced the aggressiveness of human MLL-AF9 oncogene-induced AML in mouse transplantation models [32].

Most tumors are of a clonal origin but often exhibit heterogeneity in phenotypic and functional properties, including proliferation, morphology, motility, and differentiation. Such heterogeneity has also been implicated in response to TGFβ. Although lower TGFβR2 mRNA levels in AML patients may be due to escaping from inhibition of TGFβ1 [32], patients with relatively higher TGFβR2 levels acquired poorer OS that may attribute to stronger invasiveness and drug resistance ability. For example, liver cancer cells that survive the apoptotic effects of TGFβ undergo epithelial-to-mesenchymal transition (EMT), making them resistant to cell death and prone to acquire invasive properties [33]. Therefore, we speculated that TGFβ-sensitive AML cells that can survive the inhibitory effect of TGFβ might acquire stronger malignancy at the expense of proliferation and tumor growth. In our in vitro experiments, we observed a slight inhibition in the proliferation of KG-1a cells but followed enhanced resistance to Ara-C after TGFβ1 pretreatment. Unfortunately, the mechanism underlying this drug resistance has not yet been explored further.

In summary, our research has established an ir-lncRNAs pairs prognostic model for AML patients and speculated patients in the high-risk group were more benefit from immune checkpoint inhibitors. We also found that the TGFβ pathway was enriched in high-risk patients, and AML cell lines got more malignancy after TGFβ1 pretreatment in vitro. There are, of course, shortcomings in our study. First, we used only public data to validate the availability, so this new model urgently needs to be implemented in clinical practice. Secondly, our study was limited to observing the phenomenon, and further research is needed to understand the mechanisms underlying drug resistance to TGFβ1.

### Supplementary Information

Below is the link to the electronic supplementary material.

Supplementary file1 (XLSX 27 kb)

## Data Availability

The datasets presented in this study can be found in online repositories and be available from the corresponding author on reasonable request. The names of the repository/repositories and accession number(s) can be found in the article.

## References

[1] Ravandi F, Pierce S, Garcia-Manero G (2020). Salvage therapy outcomes in a historical cohort of patients with relapsed or refractory acute myeloid Leukemia. Clin Lymphoma Myeloma Leuk.

[2] Mendez LM, Posey RR, Pandolfi PP (2019). The interplay between the genetic and immune landscapes of AML: mechanisms and implications for risk stratification and therapy. Front Oncol.

[3] Rey J, Fauriat C, Kochbati E (2017). Kinetics of cytotoxic lymphocytes reconstitution after induction chemotherapy in elderly AML patients reveals progressive recovery of normal phenotypic and functional features in NK cells. Front Immunol.

[4] Bindea G, Mlecnik B, Angell HK (2014). The immune landscape of human tumors: implications for cancer immunotherapy. Oncoimmunology.

[5] Xu S, Wang Q, Kang Y (2020). Long noncoding RNAs control the modulation of immune checkpoint molecules in cancer. Cancer Immunol Res.

[6] Xu J, Shi A, Long Z (2018). Capturing functional long non-coding RNAs through integrating large-scale causal relations from gene perturbation experiments. EBioMedicine.

[7] Wang QM, Lian GY, Song Y (2019). LncRNA MALAT1 promotes tumorigenesis and immune escape of diffuse large B cell lymphoma by sponging miR-195. Life Sci.

[8] Liu CY, Guo HH, Li HX (2021). Identification of the 7-lncRNA signature as a prognostic biomarker for acute myeloid Leukemia. Dis Markers.

[9] Pan JQ, Zhang YQ, Wang JH (2017). lncRNA co-expression network model for the prognostic analysis of acute myeloid leukemia[J]. Int J Mol Med.

[10] Zheng Z, Wu W, Lin Z (2021). Identification of seven novel ferroptosis-related long non-coding RNA signatures as a diagnostic biomarker for acute myeloid leukemia. BMC Med Genom.

[11] Shen Y, Peng X, Shen C (2020). Identification and validation of immune-related lncRNA prognostic signature for breast cancer. Genomics.

[12] Xu Q, Wang Y, Huang W (2021). Identification of immune-related lncRNA signature for predicting immune checkpoint blockade and prognosis in hepatocellular carcinoma. Int Immunopharmacol.

[13] Wang J, Shen C, Dong D (2021). Identification and verification of an immune-related lncRNA signature for predicting the prognosis of patients with bladder cancer. Int Immunopharmacol.

[14] Batlle E, Massague J (2019). Transforming growth factor-beta signaling in immunity and cancer. Immunity.

[15] Bhagyaraj E, Ahuja N, Kumar S (2019). TGF-beta induced chemoresistance in liver cancer is modulated by xenobiotic nuclear receptor PXR[J]. Cell Cycle.

[16] Zhang Y, Zhang Y, Geng L (2016). Transforming growth factor beta mediates drug resistance by regulating the expression of pyruvate dehydrogenase kinase 4 in colorectal cance. J Biol Chem.

[17] Brown JA, Yonekubo Y, Hanson N (2017). TGF-beta-induced quiescence mediates chemoresistance of tumor-propagating cells in squamous cell carcinoma. Cell Stem Cell.

[18] Tyner JW, Tognon CE, Bottomly D (2018). Functional genomic landscape of acute myeloid leukaemia. Nature.

[19] Morad G, Helmink BA, Sharma P (2021). Hallmarks of response, resistance, and toxicity to immune checkpoint blockade. Cell.

[20] Roma-Rodrigues C, Mendes R, Baptista P V, et al. Targeting Tumor Microenvironment for Cancer Therapy[J]. Int J Mol Sci, 2019,20(4). 10.3390/ijms20040840 PMC6413095 30781344

[21] Chen C, He W, Huang J (2018). LNMAT1 promotes lymphatic metastasis of bladder cancer via CCL2 dependent macrophage recruitment. Nat Commun.

[22] Cui Y, Guo G. Immunomodulatory Function of the Tumor Suppressor p53 in Host Immune Response and the Tumor Microenvironment[J]. Int J Mol Sci, 2016,17(11). 10.3390/ijms17111942 PMC5133937 27869779

[23] Sharma P, Hu-Lieskovan S, Wargo JA (2017). Primary, adaptive, and acquired resistance to cancer immunotherapy. Cell.

[24] Goodman AM, Castro A, Pyke RM (2020). MHC-I genotype and tumor mutational burden predict response to immunotherapy. Genome Med.

[25] Samstein RM, Lee CH, Shoushtari AN (2019). Tumor mutational load predicts survival after immunotherapy across multiple cancer types. Nat Genet.

[26] Rizvi NA, Hellmann MD, Snyder A (2015). Cancer immunology. Mutational landscape determines sensitivity to PD-1 blockade in non-small cell lung cancer. Science.

[27] Robey RW, Pluchino KM, Hall MD (2018). Revisiting the role of ABC transporters in multidrug-resistant cancer. Nat Rev Cancer.

[28] Levantini E, Maroni G, Del RM (2022). EGFR signaling pathway as therapeutic target in human cancers. Semin Cancer Biol.

[29] Germain N, Dhayer M, Boileau M, et al. Lipid Metabolism and Resistance to Anticancer Treatment. Biology (Basel), 2020,9(12). 10.3390/biology9120474 PMC7766644 33339398

[30] Fukuda Y, Lian S, Schuetz JD (2015). Leukemia and ABC transporters. Adv Cancer Res.

[31] Keller JR, Sing GK, Ellingsworth LR (1989). Transforming growth factor beta: possible roles in the regulation of normal and leukemic hematopoietic cell growth. J Cell Biochem.

[32] Krause DS, Fulzele K, Catic A (2013). Differential regulation of myeloid leukemias by the bone marrow microenvironment. Nat Med.

[33] Franco DL, Mainez J, Vega S (2010). Snail1 suppresses TGF-beta-induced apoptosis and is sufficient to trigger EMT in hepatocytes. J Cell Sci.

